# Regulatory domains controlling high intestinal vitamin D receptor gene expression are conserved in mouse and human

**DOI:** 10.1016/j.jbc.2022.101616

**Published:** 2022-01-21

**Authors:** James C. Fleet, Dennis Aldea, Lei Chen, Sylvia Christakos, Michael Verzi

**Affiliations:** 1 Department of Nutritional Science, University of Texas, Austin, Texas, USA; 2 Department of Genetics, Rutgers University, New Brunswick, New Jersey, USA; 3 New Jersey Medical School, Rutgers, The State University of New Jersey, Newark, New Jersey, USA

**Keywords:** vitamin D, vitamin D receptor, steroid hormone receptor, transcription, transcription factor, transcription enhancer, intestinal epithelium, colon, small intestine, genomics, 1,25(OH)2D, 1,25 dihydroxyvitamin D, ATAC-Seq, assay for transposase-accessible chromatin using sequencing, CDX2, caudal type homeobox 2, ChIP, chromatin immunoprecipitation, ENCODE, Encyclopedia of DNA Elements, GATA4, GATA-binding protein 4, GEO, Gene Expression Omnibus, H3K4me3, trimethylated histone H3 on lysine 4, HNF4a/g, hepatocyte nuclear factor 4a/g, IGV, Integrative Genomics Viewer, SMAD4, Small worm phenotype and Mothers Against Decaperntaplegic 4, TAD, topologically associated domain, TF, transcription factor, TFBS, TF-binding site, VDR, vitamin D receptor

## Abstract

Vitamin D receptor (VDR) levels are highest in the intestine where it mediates 1,25 dihydroxyvitamin D-induced gene expression. However, the mechanisms controlling high intestinal VDR gene expression are unknown. Here, we used Assay for Transposase-Accessible Chromatin using Sequencing (ATAC-Seq) to identify the regulatory sites controlling intestine-specific *Vdr* gene expression in the small intestine (villi and crypts) and colon of developing, adult, and aged mice. We identified 17 ATAC peaks in a 125 kb region from intron 3 to −55.8 kb from exon 1 of the *Vdr* gene. Interestingly, many of these peaks were missing/reduced in the developing intestine. Chromatin ImmunoPrecipitation-Sequencing (ChIP-Seq) peaks for intestinal transcription factors (TFs) were present within the ATAC peaks and at HiChIP looping attachments that connected the ATAC/TF ChIP peaks to the transcription start site and CCCTF-binding factor sites at the borders of the *Vdr* gene regulatory domain. Intestine-specific regulatory sites were identified by comparing ATAC peaks to DNAse-Seq data from other tissues that revealed tissue-specific, evolutionary conserved, and species-specific peaks. Bioinformatics analysis of human DNAse-Seq peaks revealed polymorphisms that disrupt TF-binding sites. Our analysis shows that mouse intestinal *Vdr* gene regulation requires a complex interaction of multiple distal regulatory regions and is controlled by a combination of intestinal TFs. These intestinal regulatory sites are well conserved in humans suggesting that they may be key components of VDR regulation in both mouse and human intestines.

Vitamin D is a nutrient that serves as the precursor for 1,25 dihydroxyvitamin D (1,25(OH)_2_D), a hormone that controls a wide variety of biological processes relevant to human health (1). The actions of 1,25(OH)_2_D are mediated through the induction of gene transcription following activation of the vitamin D receptor (VDR)—a ligand-activated transcription factor (TF) (2) The most studied action of vitamin D is the control of intestinal calcium absorption (3, 4). As such, it is not surprising that the VDR protein was first identified in the chick intestine as a high-affinity 1,25(OH)_2_D-binding protein (5). Since then, VDR protein and gene expression has been identified in many different tissues (6), but the highest expression of VDR is seen in the intestinal epithelium (7, 8). Consistent with a critical role for 1,25(OH)_2_D-mediated gene expression in the intestine, Lee *et al.* (9) showed that there are several thousand vitamin D-inducible DNA-binding sites for VDR and more than 600 vitamin D-regulated genes in the proximal intestine of mice.

A number of groups have identified conditions that regulate the intestinal expression of the VDR gene, including induction by glucocorticoids (10) and estrogens (11), as well as developmentally induced expression that occurs in the late postnatal period (12, 13) and declines that occur with aging (14, 15). Yet despite the critical role that VDR plays in intestinal biology, very little research exists to explain the mechanism for high intestinal *Vdr* gene expression.

Here, we report the results from primary assay for transposase-accessible chromatin using sequencing (ATAC-Seq) and Hi-chromatin immunoprecipitation (ChIP) data to identify the active regulatory domain of the mouse *Vdr* gene. We then integrated these data with publicly available ChIP-Seq data combined with TF-binding site (TFBS) analysis of these regulatory regions to define the critical TFs controlling mouse intestinal *Vdr* gene expression. In addition, we compared our data to DNAse-Seq or ATAC-Seq data from other mouse tissues and human small intestine to identify the tissue-specific and evolutionarily conserved regulatory regions controlling human intestinal *VDR* gene expression. These results provide a clear picture of the regulatory complexity controlling *Vdr* gene expression in the intestine.

## Results

**Figure 1:**
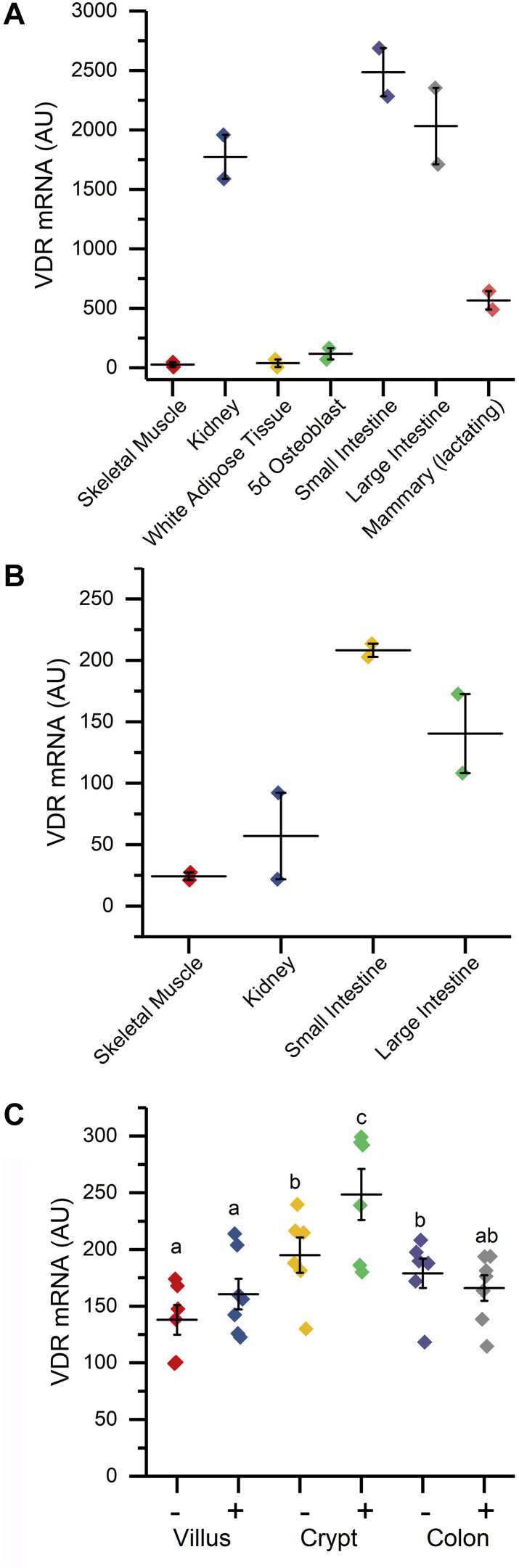
**Expression of *Vdr* mRNA in various tissues.***A*, mouse from BioGPS. *B*, human from BioGPS (16). The duplicate sample values for the tissue are presented. See Figs. S1–S3 for a complete list of tissues and cells. *C*, *Vdr* mRNA levels in the small intestine crypt, small intestine villus, and colon of vehicle and 1,25(OH)_2_D-treated (10 ng/g body weight, 4 h) vitamin D-deficient mice. Data are shown as individual data points along with means ± standard deviation (n = 6–7). Groups with different letter superscripts are significantly different from one another (*p* < 0.05). 1,25(OH)_2_D, 1,25 dihydroxyvitamin D; Vdr, vitamin D receptor.

Data from BioGPS (16) show that the intestine has the highest *Vdr* gene expression in both mouse (Fig. 1*A*) and human (Fig. 1*B*). Full tissue/cell profiles from BioGPS and the Immunological Genome Project (ImmGen (17)) are available in Figs. S1–S3. Although the intestine has high *Vdr* gene expression, we previously reported that *Vdr* mRNA levels are similar across the various segments of adult mouse intestine (18). In mice injected with vehicle, *Vdr* mRNA expression was about 30% higher in small intestine crypt and colon than small intestine villi (*p* < 0.05, Fig. 1*C*). Consistent with other reports (7), 1,25(OH)_2_D treatment had either no effect (in small intestine villi, colon) or a modest impact (20% higher in small intestine crypt) on *Vdr* mRNA levels in the intestine.

**Figure 2:**
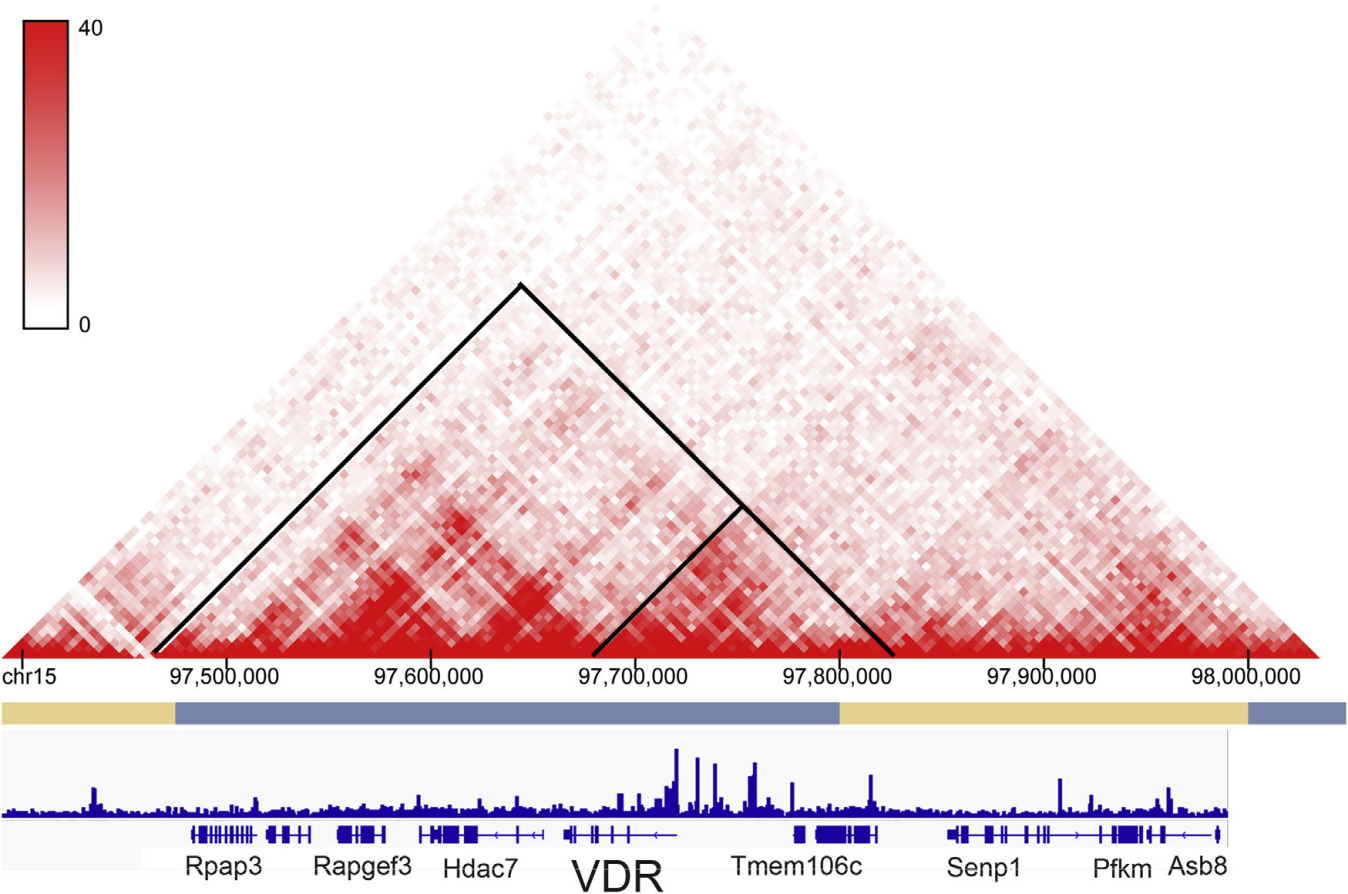
**The mouse *Vdr* gene sits within a large regulatory domain.** Hi-C data from CH12 cells (resolution of 5 kb) were obtained and visualized at the 3Dgenome.org (20). These data show that the *Vdr* gene lies within a topologically associated domain containing nine genes and several subdomains (outlined in *black triangles*). The *Vdr* gene ATAC peaks from the villus of 90-day-old mouse small intestine lie in a subdomain with the *Tmem106c* gene. ATAC, assay for transposase-accessible chromatin; Vdr, vitamin D receptor.

**Figure 3:**
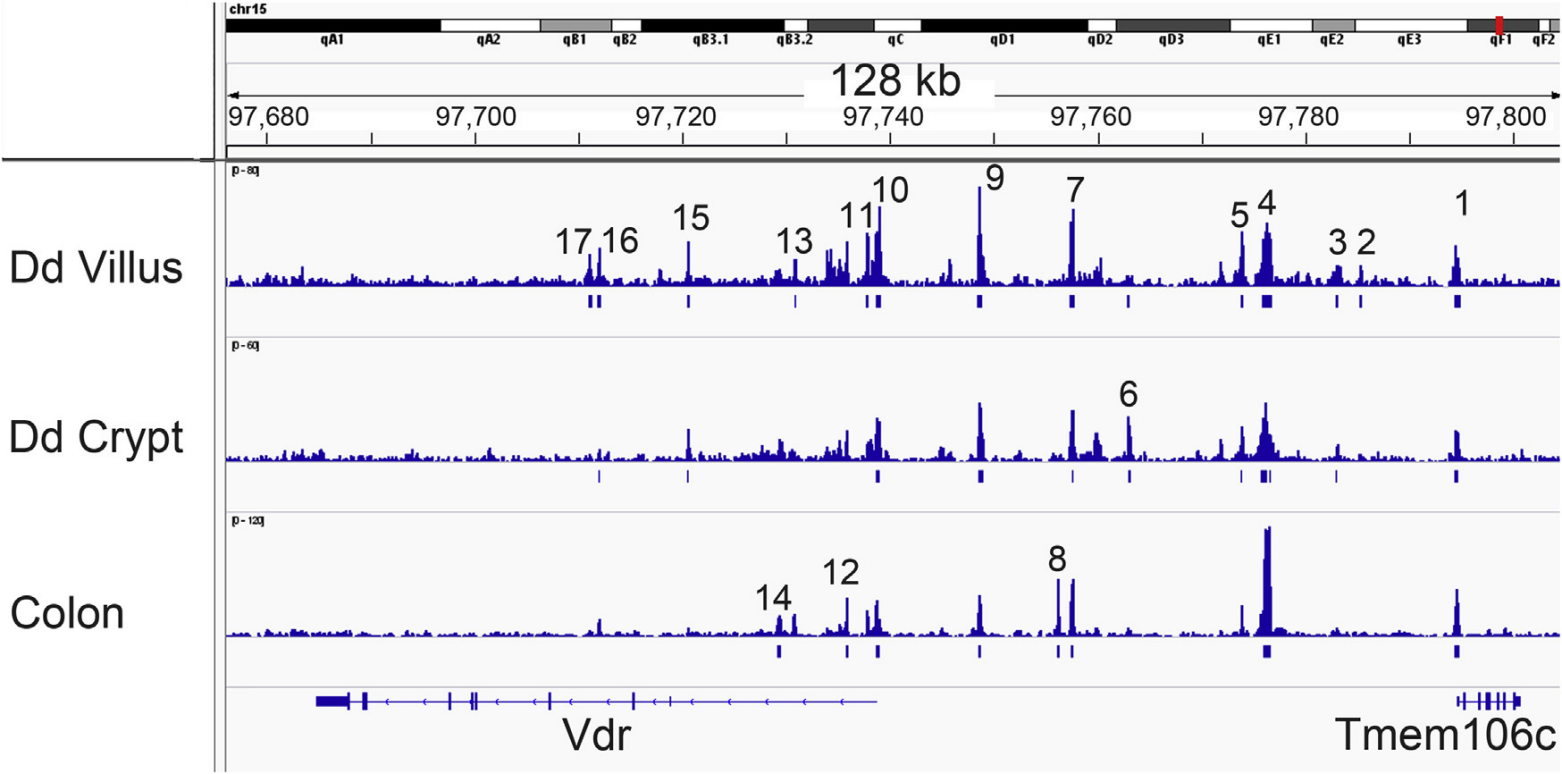
**Multiple regions control of intestinal expression of the mouse *Vdr* gene.** ATAC data from small intestine crypt and villus or colon of 90-day-old mice show that the topologically associated domain for the mouse *Vdr* gene contains multiple unique regions of accessible DNA. The majority of the ATAC peaks are seen in all three segments. ATAC, assay for transposase-accessible chromatin; Vdr, vitamin D receptor.

Regulatory regions for genes are organized into wide topologically associated domains (TADs) that contain multiple genes, span many kilobases, and are well conserved across tissues and cells within the same species (19). To define the regulatory region within the mouse *Vdr* gene TAD, we examined how the ATAC peaks we generated from the small intestine villi related to Hi-C data from CH12 cells (20). This analysis indicates that although the *Vdr* gene TAD spans a region of more than 300,000 kb and contains seven genes (*Rpap3*, *Endou*, R*apgef3*, *Slc48a1*, *Hdac7*, *Vdr*, and *Tmem106*), the ATAC peaks in small intestine are within a subdomain of 128 kb that runs from the 3′ end of the *Vdr* gene to the start of the *Tmem106c* gene (Fig. 2). Within this subdomain, we identified 17 ATAC peaks across the adult small intestine crypt, small intestine villus, and colon (Fig. 3). While the peaks in the small intestine crypt and villus were not differentially enriched in this region, DiffBind analysis showed that there were four enriched peaks in the colon compared with the small intestine villus or crypt (peaks 4, 8, and 12 for both, peak 1 for crypt). Advancing age did not alter the number of peaks in the small intestine or colon (see Figs. S4–S6 for the impact of age on ATAC peaks from small intestine villus, crypt, and colon). In contrast, DiffBind analysis showed that a number of peaks were lower or absent in the neonatal intestine compared with the adult (peaks 4, 5, 7, 9, 11, 12, and 15 for small intestine; peaks 5, 9, 11, and 12 for colon).

**Figure 4:**
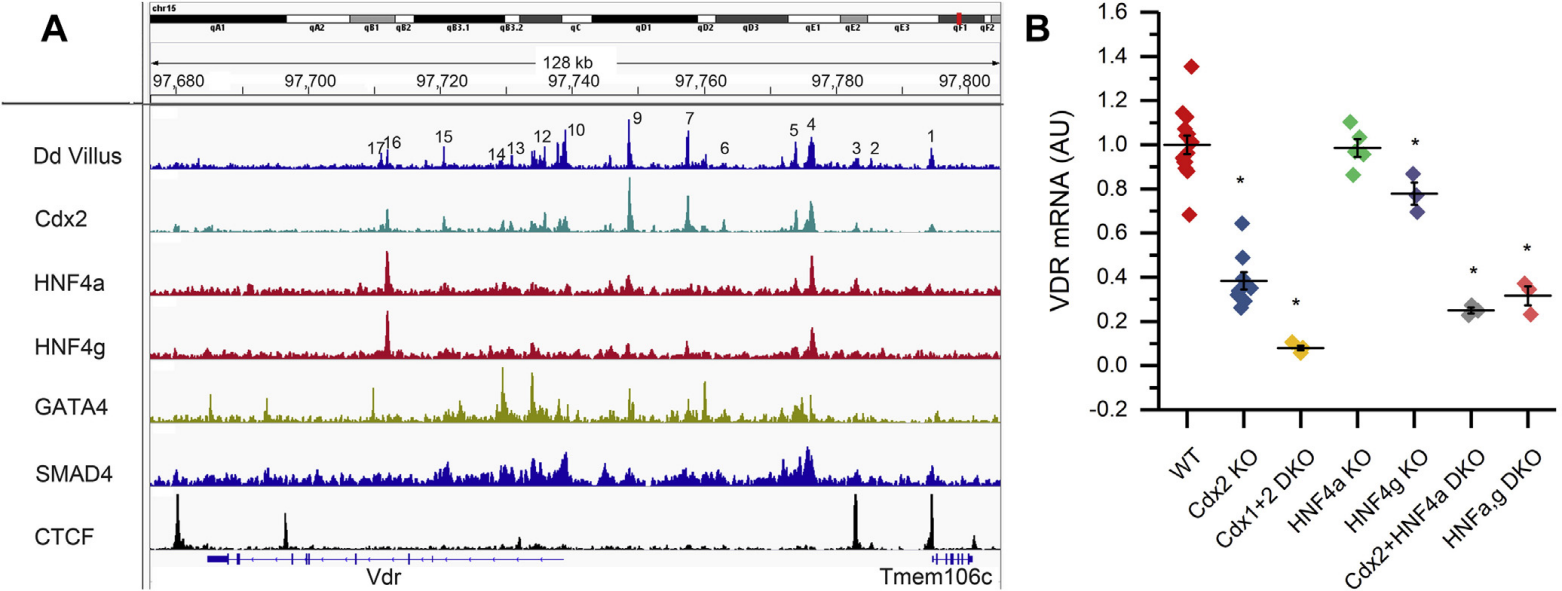
**CDX2, HNF4, GATA4, and SMAD4 bind to regulatory regions in the mouse *Vdr* gene.***A*, the ATAC-Seq peaks we identified at the *Vdr* gene locus coincide with ChIP-Seq peaks for several transcription factors known to regulate small intestinal biology. *B*, deletion of individual genes or combinations of genes for the transcription factors CDX1, CDX2, HNF4a, or HNF4g reduced *Vdr* mRNA levels in mouse small intestine. *Vdr* mRNA in WT mice is defined as 1, and expression in the various knockout lines is expressed relative to the WT level. Data are presented as individual values and means ± standard deviation for n = 3 to 14 observations. ∗*p* < 0.0083 (Bonferroni corrected) *versus* WT. ATAC-Seq, assay for transposase-accessible chromatin sequencing; CDX, caudal type homeobox 2; ChIP-Seq, chromatin immunoprecipitation using sequencing; GATA4, GATA-binding protein 4; HNF, hepatocyte nuclear factor; Vdr, vitamin D receptor.

**Figure 5:**
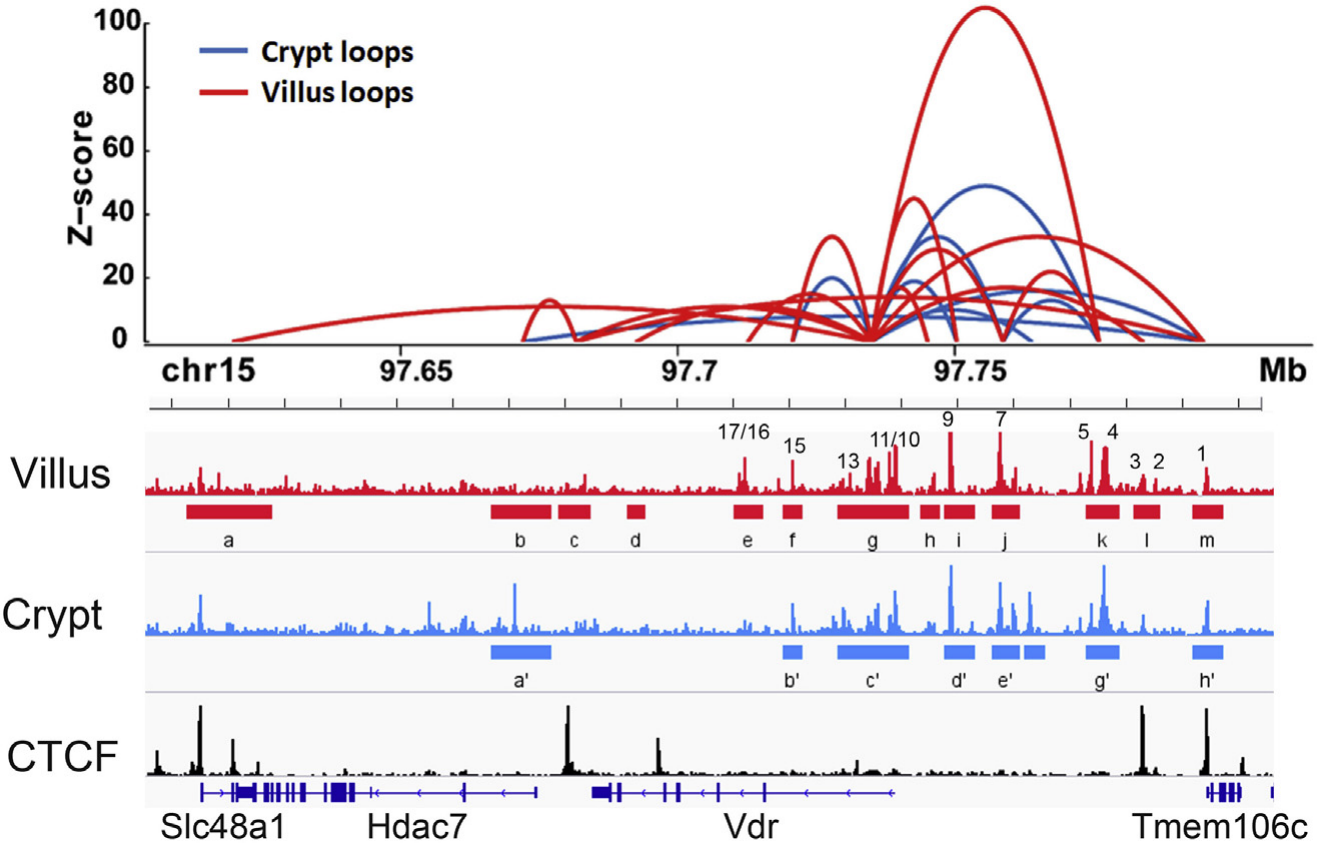
**DNA looping shows the importance of specific ATAC peaks and CTCF sites for *Vdr* gene regulation in mouse intestine.** In small intestine, 14 villus and 12 crypt ATAC-Seq peaks at the mouse *Vdr* gene locus are involved in regulatory looping. In addition, contact points at the ends of the regions correspond to the location of CTCF sites that define the ends of the regulatory domain. *Bars* under the ATAC peak data reflect the points of looping contact in villus epithelial cells (labeled a–m) or crypt epithelial cells (labeled a'–h'). ATAC, assay for transposase-accessible chromatin; CTCF, CCCTF-binding factor; Vdr, vitamin D receptor.

Figure 4*A* shows that ChIP-Seq peaks for multiple intestine-specific or enriched TFs (*i.e.*, caudal type homeobox 2 [CDX2], GATA-binding protein [GATA4], hepatocyte nuclear factor 4a/g [HNF4a/g], Small worm phenotype and Mothers Against Decaperntaplegic 4 [SMAD4]) overlap with the intestinal ATAC peaks at the *Vdr* gene locus. Bioinformatics analysis identified conserved sequences for the binding sites of these TFs in many of the 17 peaks (Table S1). In particular, peaks 4 and 9 had both strong ChIP-Seq peaks and predicted binding sites for each of these TFs. This suggests that these TFs combine to control intestinal *Vdr* gene expression in mouse. Consistent with this hypothesis, intestinal *Vdr* mRNA expression levels are significantly reduced in mice lacking either CDX2, HNF4a, or HNF4g, whereas combined deletion of multiple TFs has an even greater negative impact on *Vdr* mRNA level (Fig. 4*B*). In addition to overlap of our ATAC peaks with TFBSs, peak 1 and a downstream ATAC peak near the *Slc48a1* gene overlap with CCCTF-binding factor ChIP-Seq peaks (Fig. 5) suggesting they create a regulatory loop within the mouse *Vdr* gene TAD. In support of this hypothesis, chromatin conformation studies using trimethylated histone H3 on lysine 4 (H3K4me3)-HiChIP-Seq on villus and crypt cells show that these CCCTF-binding factor sites are part of the regulatory looping controlling *Vdr* gene expression in small intestine villi (Fig. 5). The looping structures for both small intestine crypt and villus converge on peaks 10 to 14 at the transcription start site and include the upstream enhancers at peaks 4, 7, and 9 as well as a downstream enhancer at peak 15. The regulatory looping around the *Vdr* gene in the small intestine villus is more complex than in the crypt (*i.e.*, using unique peaks 2, 3, 16, and 17), whereas the crypt uses a unique contact point at peak 6 and a contact point at an enriched ATAC peak within intron 1 of the *Hdac7* gene.

**Figure 6:**
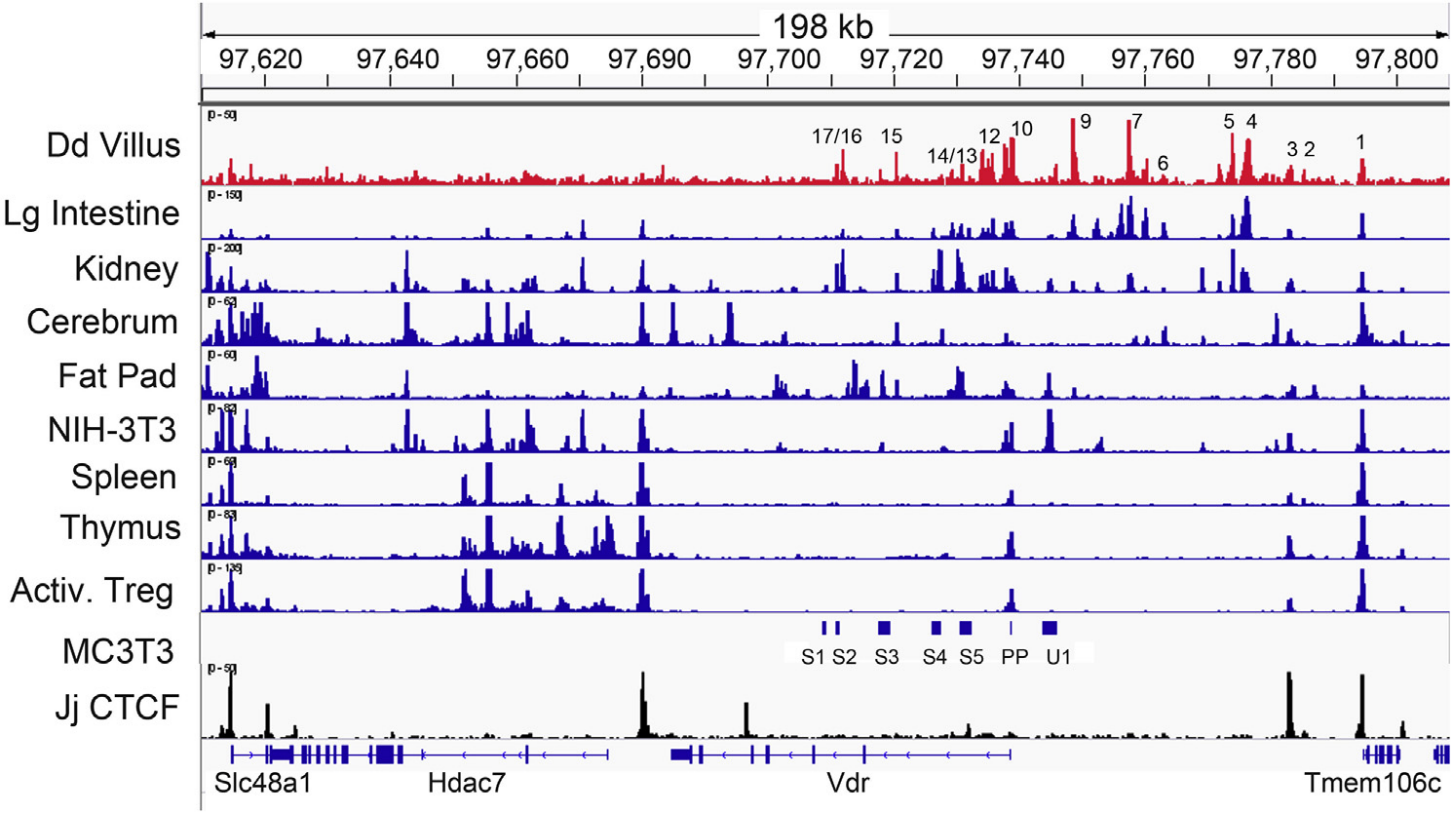
**A comparison of accessible DNA regions among mouse tissues and cells.** The *top row* of data is ATAC data from the small intestine villus epithelial cells of 90-day-old mice. DNAse-Seq from ENCODE was used to identify open chromatin regions around the *Vdr* gene in various mouse tissues/cells. Open chromatin regions previously identified in MC3T3 osteoblast cells are presented as *bars* and labeled as reported by Zella *et al.* (21). CTCF ChIP-Seq data from mouse jejunum are presented in the *bottom row*. ATAC, assay for transposase-accessible chromatin; ChIP-Seq, chromatin immunoprecipitation sequencing; CTCF, CCCTF-binding factor; ENCODE, Encyclopedia of DNA Elements; Vdr, vitamin D receptor.

We found that the intestinal ATAC-Seq peaks were similar to the DNase-Seq peaks seen for mouse large intestine in the Encyclopedia of DNA Elements (ENCODE) project (Fig. 6). Renal *Vdr* mRNA expression is also high in the mouse (Fig. 1*A*) and kidney DNase-Seq peaks from ENCODE overlapped the intestinal ATAC peaks within and upstream of the *Vdr* gene at peaks 1, 3 to 5, 7, and 9 to 17. However, the relative intensity of peaks is different in the kidney (lower peaks 7 and 9; higher peaks 13 and 14), there were three additional peaks (between intestinal peaks 5 and 6; between peaks 9 and 10; and between peaks 14 and 15), and there were more kidney DNase-Seq peaks downstream from the *Vdr* gene where the intestine has no peaks. In contrast, DNase-Seq data from other mouse tissues (Fig. 6) and ATAC-Seq data on mouse immune cells from ImmGenn (Fig. S7) revealed that most tissues lack the peaks seen in intestine, and only peaks 10 and 11 at the transcription start site were seen across cells and tissues. Zella *et al.* (21) previously identified seven regulatory regions controlling *Vdr* gene expression in mouse, MC3T3 osteoblast-like cells (S1–S5, PP, and U1). Three of these overlapped with intestinal ATAC peaks (*i.e.*, S2 = peak 17, S4 = peak 14, and PP = peak 10). In addition, the U1 region from Zella *et al.* was at a looping contact point seen in the small intestine villi, despite the fact that Model-based Analysis of ChIP-seq 2 analysis of the intestinal ATAC data did not detect a peak in this region.

**Figure 7:**
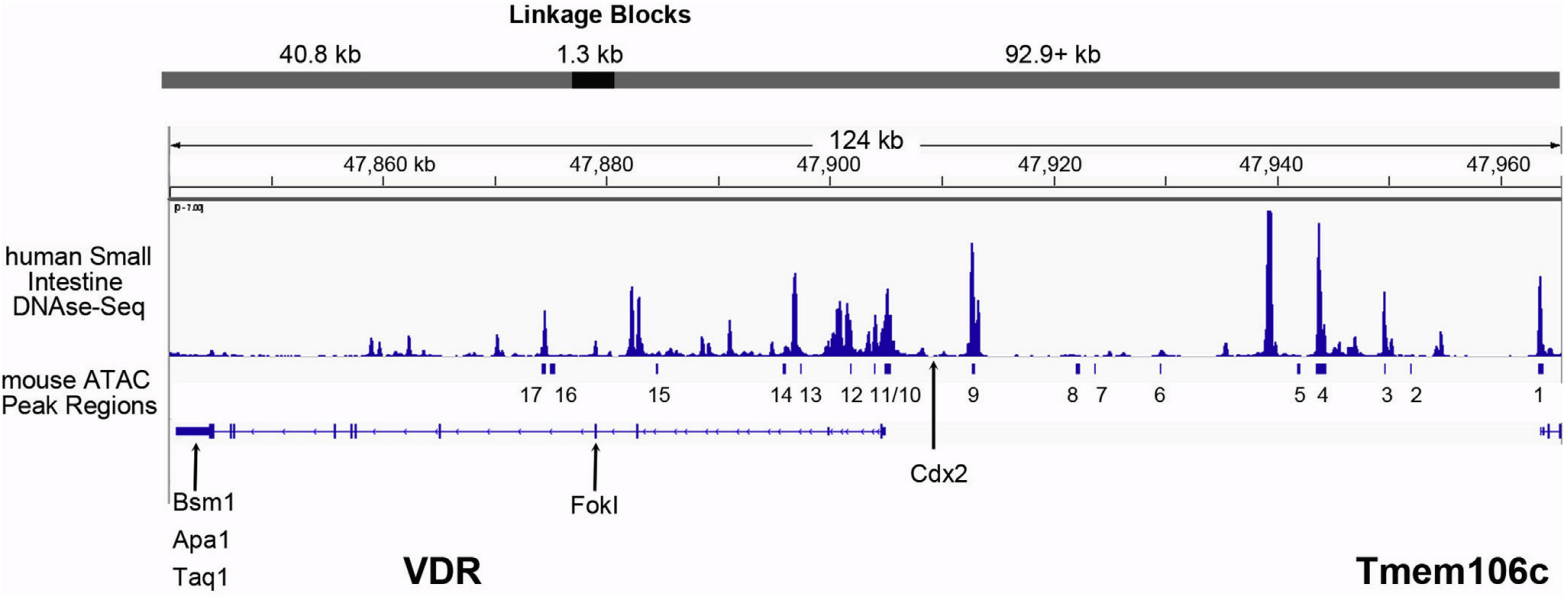
**A comparison of regulatory peaks between mouse and human at the *VDR* gene locus.** Small intestine DNAse-Seq data from the human ENCODE project were compared with the mouse intestinal ATAC-Seq peak sequences using the Matcher program in EMBOSS. *Bars* under the human DNAse-Seq data line reflect the areas of the *VDR* gene that are homologous to the mouse *Vdr* gene ATAC-Seq peaks. Locations of common *VDR* gene polymorphisms are identified with *arrows* (FokI, Bsm1, Apa1, Taq1 restriction fragment length polymorphisms; CDX2 transcription factor–binding site polymorphism). Blocks where DNA variation is in linkage disequilibrium (44) are identified at the *top* of the graphic. ATAC-Seq, assay for transposase-accessible chromatin using sequencing; CDX2, caudal type homeobox 2; ENCODE, Encyclopedia of DNA Elements; VDR, vitamin D receptor.

To determine whether the mouse regulatory domains are conserved through evolution into humans, we identified human VDR gene sequences that were homologous to our mouse intestinal ATAC peaks and then examined whether these conserved regions overlapped with DNase-Seq peaks from the adult human small intestine. Although the spacing of peaks was different in human, eight of the human VDR DNase-Seq peaks had significant sequence homology with the mouse ATAC peaks (*i.e.*, peaks 1, 3, 4, 9, 10, 11, 12, and 17), whereas mouse peaks 2, 5, 7, 13, 14, 15, and 16 were absent in the human *VDR* gene (Fig. 7). In addition, there were at least six unique peaks in the human *VDR* gene that were not seen in mouse. Bioinformatics analysis of the sequences in the *VDR* gene that are underneath the shared and unique peaks shows that binding sites for CDX2, HNF4a/g, GATA4, and SMAD4 are present in these regions (Table S2). Similar to what we found in the mouse, human peaks corresponding to mouse peaks 4 and 9 had conserved binding sites for all four TFs.

**Figure 8:**
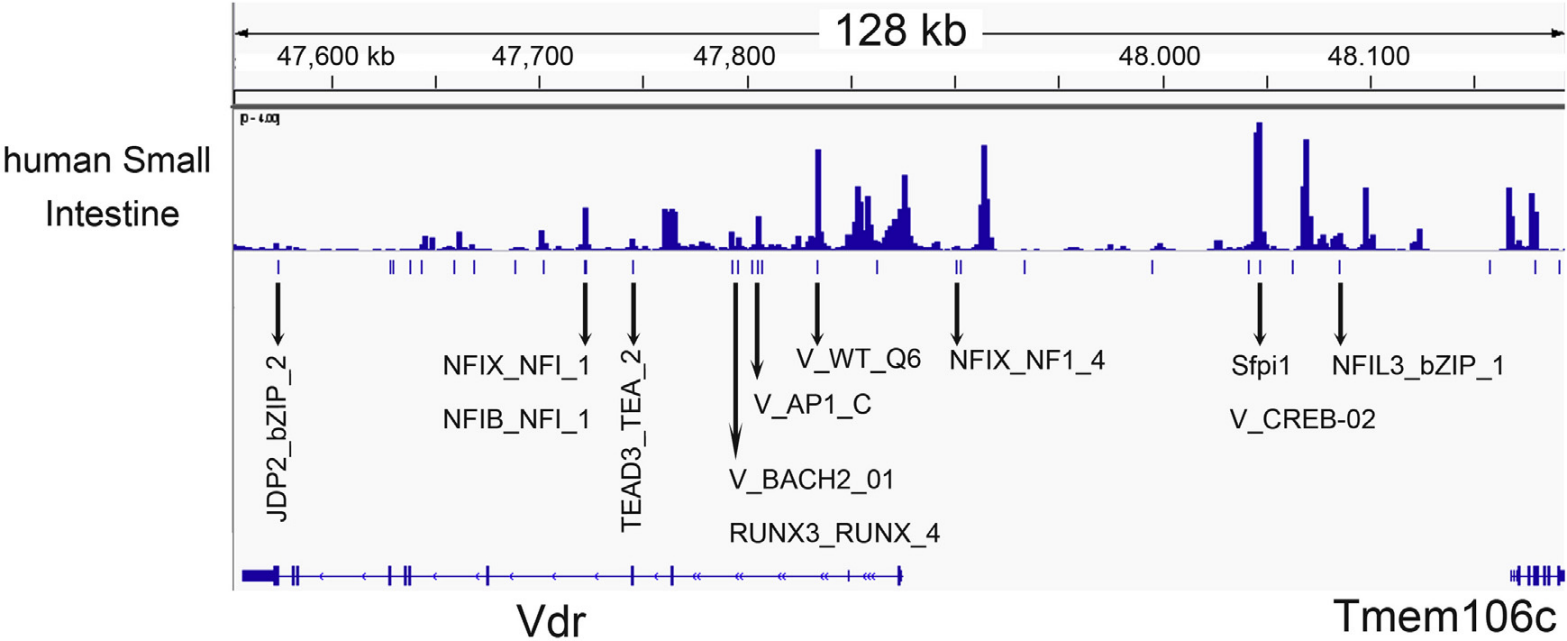
**Human *VDR* gene regulatory peaks contain polymorphisms predicted to disrupt transcription factor–binding sites.** Human small intestine DNAse-Seq data from human ENCODE were integrated with a set of common genetic variants predicted to directly influence transcription factor occupancy and regulatory DNA accessibility from Maurano *et al.* (22). *Bars* under the DNAse-Seq data represent the transcription factor–binding sites predicted to be affected at the *VDR* gene locus. *Arrows* identify specific binding sites that are within human small intestinal DNAse-Seq peaks. ENCODE, Encyclopedia of DNA Elements; VDR, vitamin D receptor.

Maurano *et al.* (22) previously used ENCODE data to identify more than 60,000 common genetic variants that directly influence TF occupancy and regulatory DNA accessibility in the human genome. By intersecting these variant data with the human DNase-Seq peaks, we identified 12 TFBSs whose function are compromised by common genetic variation present in the human genome (Fig. 8), including sites for several TFs known to regulate gene expression in the intestine (*i.e.*, NFIB, NFIX, NFIL3, SFPI1, RUNX3, TEAD3). No variants affected the TFBS for Cdx2, HNF4, GATA4, or SMAD4.

## Discussion

Our data clearly show that the high-level expression of VDR in the intestine is controlled by a regulatory network of TFs acting at multiple upstream and downstream DNA sequences. Few studies have examined how the *Vdr* gene is regulated, but our data identified 15 ATAC peaks that are involved in regulatory loops and control intestinal VDR gene expression in the mouse (Fig. 5). In addition, we found that at least four TFs control this regulation in the small intestine: CDX2, HNF4g, GATA4, and SMAD4 (Fig. 4*A*). Others have previously shown that these TFs have a central role in small intestine–specific gene expression; for example, Vierstra *et al.* (23) found that binding motifs for CDX2, HNF4a/g, and GATA 4/5/6 were enriched in 4575 ATAC-Seq peaks that are seen in intestine but not in other tissues. Among these, CDX2 is viewed as a master regulator of intestinal epithelial cell identity (24) that maintains a permissive chromatin environment that favorably influences the DNA binding of other TFs, for example, the intestine restricted TF HNF4a (25). Similarly, SMAD4 and HNF4 activate each other's expression and cobind to regulatory elements of intestinal differentiation genes to stabilize enterocyte identity (26), whereas other evidence shows that GATA4 is critical for gene expression in the proximal small intestine (27). However, the established role for these TFs neither precludes a role for other TFs in the small intestine nor does explain the regulation in the colon, where GATA4 is absent (28). The small intestine and colon express more than 300 TFs, of which about 100 are tissue restricted and likely to be important for intestine-specific gene expression (29). Future studies should examine other TFs that may contribute to mouse *Vdr* gene expression including the colon-enriched TFs GATA6, SATB2, and KLF4 and the small intestine–restricted TF PDX1.

Various groups have reported that intestinal VDR levels are increased during development and reduced by aging. Pierce and DeLuca (13) observed low VDR protein levels up to 15 days postnatal in rat pups that then increased by 21 days postnatal, a point that coincides with the development of vitamin D responsive intestinal calcium absorption (12). In addition, at about 2 weeks after birth, the rodent intestine undergoes major structural and functional changes reflecting maturation and development of the ability to digest solid food (30). This coincides with the intestinal sensitivity to glucocorticoids (31), a treatment that induces intestinal development and increases intestinal VDR levels (12). Our ATAC data show that many important regulatory sites seen in the adult intestine, including peaks 4 and 9 that bind CDX2, HNF4, GATA4, and SMAD4, are absent in 12 days postnatal mouse intestine. Thus, our data show that essential regulatory domains controlling *Vdr* gene expression are not accessible until after the intestine develops the ability to digest solid food.

In contrast to the effects of development, our data show that aging has a modest impact on the accessibility of intestinal *Vdr* gene regulatory sites. Several groups previously reported that aging reduces basal and vitamin D-dependent intestinal calcium absorption in humans (32) and rodents (33) and that this may be due to a reduction in intestinal VDR levels as seen in rats (15) and adult women (34). Consistent with this, we have reported that the intestinal response to 1,25(OH)_2_D is sensitive to changes in VDR level (35, 36). However, we previously reported that intestinal VDR levels are stable across the life span of rats (33), and our new data show that the regulatory peaks controlling VDR gene expression (Figs. S4–S6) do not change in adult mice. Collectively, while age-associated intestinal resistance to vitamin D action is a real phenomenon, our data suggest that it is not because of age-associated differences in *Vdr* gene regulation but depends upon other factors that influence VDR function at target genes.

In addition to the intestine, many other cells and tissues express VDR (6). To gain insight into the tissue-specific regulatory sites controlling intestinal VDR gene expression, we compared our intestinal ATAC peaks to published data from MC3T3 osteoblast-like cells (21), publicly available DNAse-Seq data from ENCODE, and ATAC-Seq from the Immunological Genome project (ImmGen.org). As expected, ENCODE data on the mouse large intestine are very similar to our novel ATAC-Seq analysis of small intestinal crypts and villi as well as from the colon. The DNA accessibility at the intestinal ATAC-Seq peaks was silent for most other cells and tissues—indicating their expression is controlled by different regulatory domains. However, many of the intestine-ATAC peaks were also found in the DNAse-Seq data from kidney. The kidney DNAse-Seq peaks also overlapped with peaks Zella *et al.* (21) found to be important in MC3T3 cells: U1, PP, S1, and S3–S5. VDR protein and mRNA is expressed in the distal renal tubule segments of the distal collecting tubule, connecting tubule, cortical collecting duct, and cortical thick ascending limb (37, 38). These segments neither express the TFs controlling intestinal VDR expression (*i.e.*, CDX2, HNF4a/g, GATA4) nor do they express C/EBPb (29), a TF that binds regions U1, PP, S1, and S3–S5 in MC3T3 (39). In the kidney, the regulatory sites identified by DNAse-Seq are more likely to bind to renal-enriched TFs like HNF1a, GATA3, TOX3, MECOM, HOXD10, or HOXB7, whose expressions are highest in the distal renal tubule segments (38).

We compared the DNA sequences under our mouse intestinal ATAC peaks to the sequence of the human *VDR* gene and found that there are evolutionarily conserved regulatory sites (mouse peaks 1, 3, 4, 9, 10–12, and 17) and those that are unique to the human (*i.e.*, a large peak between mouse peaks 5 and 6; and peaks between mouse peaks 13 and 14 or between peaks 15 and 16). Based on our bioinformatics analysis of human DNAse-Seq peaks (Table S2), the major TFs controlling human *VDR* gene expression in the small intestine are likely to be the same as those used in mouse, for example, the conserved peaks equivalent to mouse peaks 4 and 9, as well as the large human intestine–specific peak downstream from conserved peak 4, all have well-conserved motifs for all four intestinal TFs: CDX2, GATA4, HNF4g, and SMAD4. However, no ChIP-Seq data on intestine-specific TFs exist for human small intestine or colon, and so the identity of the TF that bind to the major human DNAse-Seq peaks requires experimental validation.

A number of studies have associated polymorphisms in the VDR gene with specific health outcomes, but only one of these polymorphisms is predicted to be specific to the intestine—a variant within a binding site for the intestine-specific TF CDX2 (40). Yamamoto *et al.* (41) used EMSA and reporter gene assays to identify a putative CDX2-binding site between −3720 and −3731 upstream of the human *VDR* gene that they proposed would control intestinal VDR gene expression in humans. This group later reported that a common polymorphism could disrupt CDX2 binding to this site (40). Since then, others have associated the CDX2 TF-binding site polymorphism with health outcomes as diverse as osteoporosis risk (42) and prostate cancer (43). However, our analysis in Figure 7 shows that there is no DNAse-Seq peak over the putative CDX2-binding site (Fig. 7), suggesting that this site, while functional in *ex vivo* assays, does not have a role in *VDR* gene regulation *in vivo*.

The CDX2-binding site polymorphism lies within a large linkage block that is upstream of exon 2 in the human *VDR* gene (44). Thus, if the associations between the CDX2-binding site variant and health outcomes are real, they are likely because the site is in linkage disequilibrium with another polymorphism that disrupts a different TF-binding site. We examined this hypothesis by using data from Maurano *et al.* (22) on regulatory site polymorphisms predicted to affect TF occupancy. This analysis identified eight TF-binding sites within DNAse-Seq peaks of the human *VDR* gene that are disrupted by common genetic variability. Future studies will be needed to assess if any of these have functional relevance for the regulation of VDR gene expression in the intestine or any other tissue.

There are several strengths to our genomic analysis of intestinal *Vdr* gene expression. First, our analysis uses high-quality ATAC-Seq and HiChIP data at the mouse *Vdr* gene locus in a way that respects the three important axes that are usually ignored in vitamin D research: the crypt/villus axis of the small intestine, proximal/distal axis, and impact of development/aging. Second, we used publicly available data to confirm that the regulatory peaks exist and that they bind specific TFs that others have shown are important for intestine-specific gene regulation. However, the available public TF ChIP-Seq data focuses on the mouse small intestine. As such, additional research is needed to verify the applicability of the mouse small intestine data to the mouse colon or to the human small intestine. Also, additional experiments mutating specific TF-binding sites are necessary to confirm the importance of these sites in the long-distance interactions that mediate Vdr gene regulation. Finally, we have crossreferenced our mouse ATAC-Seq data with information available on DNA accessibility in human small intestine and with data on the impact of common genetic variants that disrupt TF-binding sites. Additional studies are needed to determine the functional consequence of these polymorphisms on human VDR gene expression and vitamin D action in the intestine. Nonetheless, the work reported here is valuable because it clarifies the mechanisms used to drive high-level VDR gene expression in the mouse and human intestine and because it identifies potential mechanisms that might alter individual responses to vitamin D in human populations.

## Experimental procedures

### *Vdr* mRNA levels in mouse intestine

Pups from dams fed vitamin D-deficient diet *ad libitum* for 2 to 3 weeks prior to mating, during pregnancy, and during lactation were continued on the vitamin D-deficient diet until the end of the experiment. They were exposed to a 12 h light/dark cycle, while food and water were given *ad libitum*. At 12 weeks of age, mice were treated with 1,25(OH)_2_D_3_ (10 ng/g body weight, intraperitoneal injection; Cayman Chemical Company), and tissue was harvested 4 h later (n = 6–7 per group). Animal studies were approved by the Rutgers University Animal Care and Use Committee.

Small intestine crypt and villi were isolated from the proximal 10 cm of small intestine using methods we have previously reported (45). Colonic mucosal scrapings were collected from the entire colon. Total RNA was prepared from samples using a two-phase isolation of Trizol extraction (Invitrogen) followed by purification with an RNeasy Plus Universal kit and on-column DNase digestion (Qiagen). Samples were analyzed by RNA-Seq using methods we have described previously (18). The full RNA-Seq dataset is available in Gene Expression Omnibus (GEO) as GSE133949. Significantly differentially expressed genes were defined as those with a 5% false discovery rate.

### Age-related changes in *Vdr* gene accessibility by ATAC-Seq

C57BL/6J mice were used, and small intestine or colon was harvested prior to weaning (12–15 days postnatal), at 90 days, or at 21 months of age. Epithelial cell preparations were from 12-day-old pups (small intestine) or 15-day-old pups (colon) (three replicates with tissue from six pups pooled). Small intestine epithelium from 90-day (n = 6) and 20-month mice (n = 3) was separated into villus and crypt fractions, whereas the whole unfractionated epithelium was used from colon.

Preparations of small intestine villi and crypts were isolated as we have described previously (26). A whole small intestine epithelium fraction was used for juvenile ATAC-Seq samples, whereas isolated small intestine villi and crypts were used for adult and geriatric samples. Freshly isolated cells from juvenile (combined from six pups) or adult colon (combined from two mice) were stained with the anti-CD326 (EpCAM) magnetic microbeads antibody (catalog no.: 130-105-958; Miltenyi Biotec), and stained cells were passed through a 40 μm cell strainer, collected over an MS column (catalog no.: 130-042-201; Miltenyi Biotec), and the resulting EpCAM-positive epithelial cells were used for ATAC-Seq. For geriatric proximal colon (combined from two mice), epithelial cells were treated with prewarmed 0.25% trypsin (8 min, 37 °C with vortexing), neutralized with 10% fetal bovine serum, and passed through a 40 μm cell strainer to obtain single cells for ATAC-Seq.

### 3D chromosome interactions

We have previously described the isolation of tissue and analysis of small intestine villus and crypt cells using H3K4me3-HiChIP-Seq (46). These data are available in GEO as GSE148691. Briefly, active intestinal chromatin regions were defined from our data and public data sources that empirically defined intestinal active chromatin regions, including ATAC-Seq, DNase-Seq, H3k4me3, H3K27ac, and H3K4me3 ChIP-Seq from intestinal stem, crypt, villus, or intestinal epithelial cells, including GEO datasets GSE148691, GSE112946, GSE98724, GSE51458, GSE90462, GSE57919, and GSE83394. Loops were visualized by Sushi package (version 1.20.0) (47) and shown with *q* ≤ 0.0001 and counts ≥8 (two combined biological replicates per condition).

### ATAC-Seq analysis

About 50,000 cells from each age/tissue group were used for ATAC-Seq analysis using standard procedures (48). One sample from the geriatric colon could not be processed because of low amount of DNA recovered. For other samples, the PCR-amplified libraries were purified, fragment size was selected using Pippin Prep, and sequenced on Illumina NextSeq 500 cycle mid output. The ATAC-Seq data are available in GEO as GSE134579 entry.

Nextera transpose adapter reverse complement was removed from the 3′ end of the ATAC-Seq reads using CutAdapt, version 1.9.1 (cutadapt.readthedocs.io/en/v1.9.1/). The trimmed FASTQ files were aligned to the mouse genome build mm9 using Bowtie2. The resulting SAM files were converted into BAM files, sorted, and replicate files merged in SAMtools, version 0.1.19. A BigWig file was generated for each sorted BAM file using the default parameters for the bamCoverage utility included in deeptools, version 2.4.2-4-99ec5c. Replicate BigWig files were merged using the BigWigMerge, version 2, and a combined BedGraph file was generated and then converted to BigWig files using BedGraphToBigWig version 4 and BED files were generated using the BAMtoBED utility included in bedtools, version 2.17.0. The median insert size of each BED file was determined using Picard, version 2.18.27 suite. Statistically significant enriched genomic regions (“peaks”) were identified using MACS2, version 2.1.0.20150731 with a 5′ shift of one-half the median insert length for each BED file. ENCODE-blacklisted regions and mitochondrial regions were removed from the BED file. Each peak was recentered on its summit.

BAM file and BED files for each sample were analyzed for differential enrichment across groups using DiffBind using pairwise comparisons across different ages (tissue-constant comparisons) and intestinal tissues (age-constant comparisons) (*p* < 2 × 10^−5^). The sets of significant differentially accessible peaks were written to a BED file.

### Public ChIP-Seq and RNA-Seq data

We compared our ATAC-Seq data to ChIP-Seq data for TFs known to be important for intestinal gene expression: CDX2 (GSM2610628; jejunum (49)), HNF4a and HNF4g (GSE112946; small intestine (26)), SMAD4 (GSM3477311; small intestine (26)), and GATA4 (GSM1688778; jejunum (50)). Active chromatin regions in other tissues were determined from data with DNAse I-Seq data from ENCODE (*i.e.*, large intestine, kidney, cerebrum, fat pad, NIH-3T3 cells available through the Integrative Genomics Viewer (IGV), version 2.8.6 (51)) or ATAC-Seq data on specific immune cell populations from ImmGen.org (thymic epithelial cells, Ep.MEChi.Th; bone marrow stem cells, LTHSC.34−.BM; lung alveolar macrophages [control] MF.Alv.Lu; [pIC exposed] MF.pIC.Alv.Lu.bw, B cells, B.Sp). BigWig or tdf files were visualized in IGV (version 2.8.6).

RNA-Seq data from the small intestine of wildtype and various single and double gene knockout mice were from the following GEO datasets: GSE112946 (small intestine, *Hnf4a*, *Hnf4g*, and double gene KO (26)), GSE34568 (*Cdx2*, *HNF4a*, and double gene KO (25)), GSE70766 (*Cdx2* gene KO (52)), and GSE24633 (*Cdx2*, *Cdx1 + Cdx2* double gene KO (53)). Mice in these experiments were 6 weeks old. VDR mRNA levels were normalized within each dataset to the mean value for the control/wildtype group; then all datasets were pooled and analyzed statistically using independent *t* tests with a Bonferroni correction.

### Bioinformatics search for TFBS

Sequences for the ATAC-Seq peaks in mouse or DNAse-Seq peaks in human were obtained from Ensembl and converted to FASTA files. Peak sequences were searched for TFBS using Ciider, version 0.9 (54) using a deficit threshold of 0.2 or 0.25 and the JASPAR 2020 core vertebrate sequences.

### Human regulatory region data

We compared mouse ATAC-Seq peak sequences from the villus of 90-day-old mice to the human VDR gene region (Chr 12:47,841–48,010,146) using the Matcher program in EMBOSS (55). The location of the conserved sequences was then compared with DNAse-Seq peaks around the human VDR gene locus using data from ENCODE (ENCBS853LFM) and visualized in the IGV (version 2.8.6). DNase-Seq data from the human small intestine were also compared with a list of TF-binding sites that contain genetic common variants that influence TF occupancy and regulatory DNA accessibility *in vivo* (22).

## Data availability

RNA-Seq data used to determine tissue *Vdr* content are available in GEO as GSE133949; H3K4me3-HiChIP data used to determine 3D chromosome interactions are available in GEO as GSE148691. ATAC-Seq data on the different intestinal compartments are available in GEO as GSE134579 entry. Public ChIP-Seq data that were used to integrate with our new ATAC-Seq data are available from GEO: CDX2 (GSM2610628), HNF4a and HNF4g (GSE112946), SMAD4 (GSM3477311), and GATA4 (GSM1688778). DNAse I-Seq data from ENCODE are available through the IGV (version 2.8.6), and ATAC-Seq data on specific immune cell populations were obtained from ImmGen.org. RNA-Seq data from various single and double gene knockout mice were from the following GEO datasets: GSE112946 (*Hnf4a*, *Hnf4g*, and double gene KO), GSE34568 (*Cdx2*, *HNF4a*, and double gene KO), GSE70766 (*Cdx2* gene KO), GSE24633 (*Cdx2*, *Cdx1 + Cdx2* double gene KO).

## Supporting information

This article contains supporting information.

## Conflict of interest

The authors declare that they have no conflicts of interest with the contents of this article.
