# Supplementary material for: Regulatory domains controlling high intestinal vitamin D receptor gene expression are conserved in mouse and human
Source: J Biol Chem. 2022 Jan 21;298(3):101616. doi: 10.1016/j.jbc.2022.101616 (PMC8891975; doi:10.1016/j.jbc.2022.101616)

Supplemental Figures and Tables: Fleet et al. (2021) Intestinal VDR gene regulation in Mouse


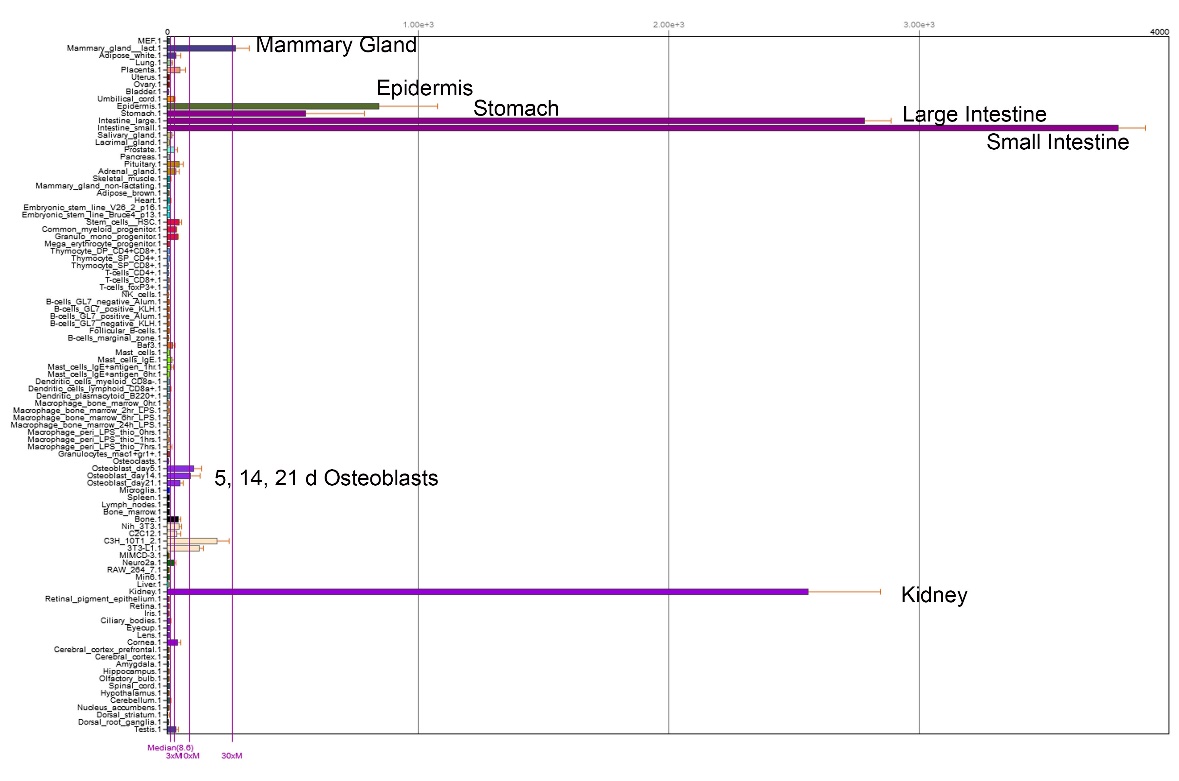


**Supplemental Figure 1S.** VDR mRNA in mouse tissues from BioGPS


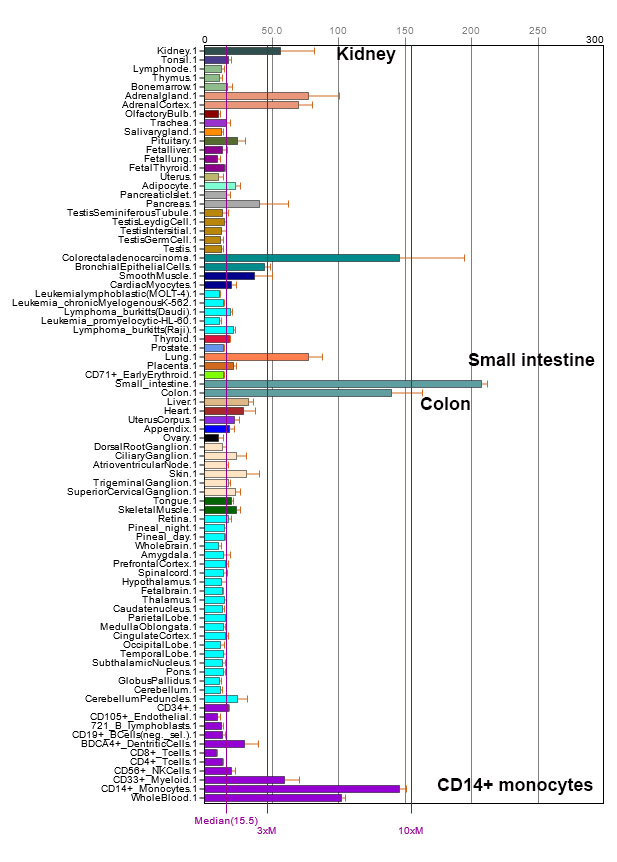


**Supplemental Figure 2S.** VDR mRNA in human tissues from BioGPS


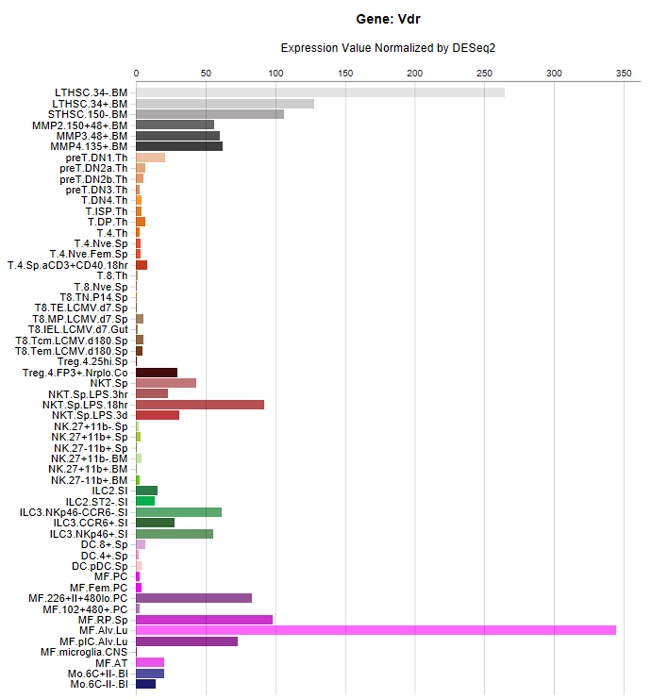


**Supplemental Figure 3S.** VDR mRNA in mouse immune cells from ImmGen


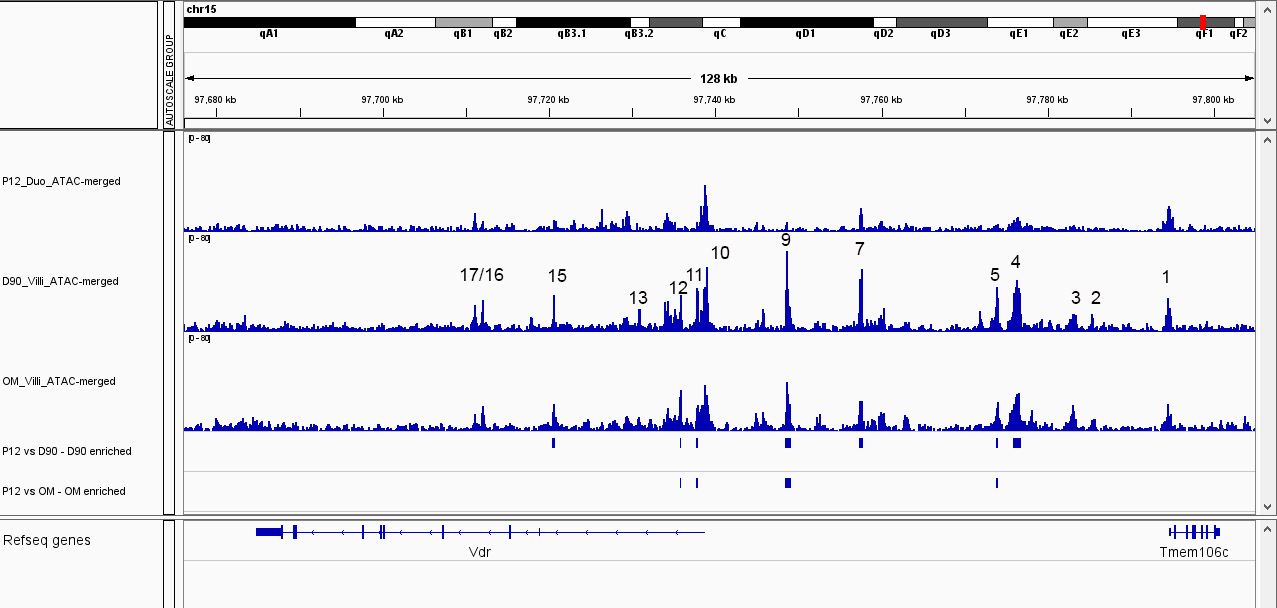


**Supplemental Figure 4S.** Effects of age on ATAC peaks in the small intestine villus. OM = old mouse


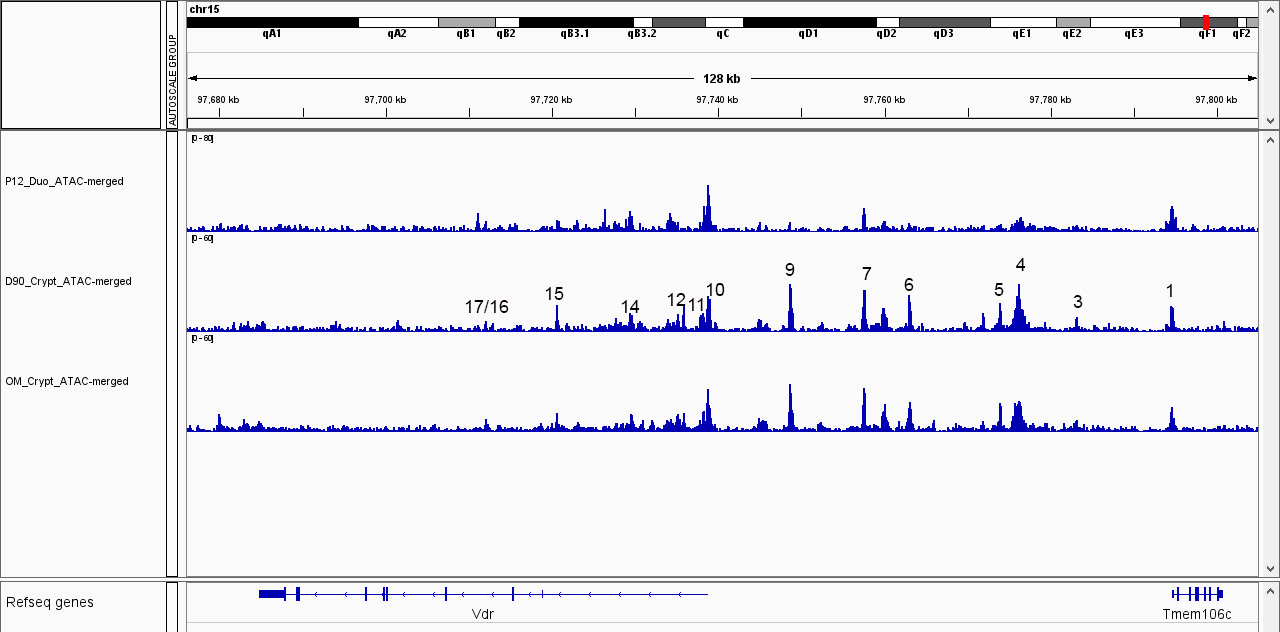


**Supplemental Figure 5S.** Effects of age on ATAC peaks in the small intestine crypts. OM = old mouse


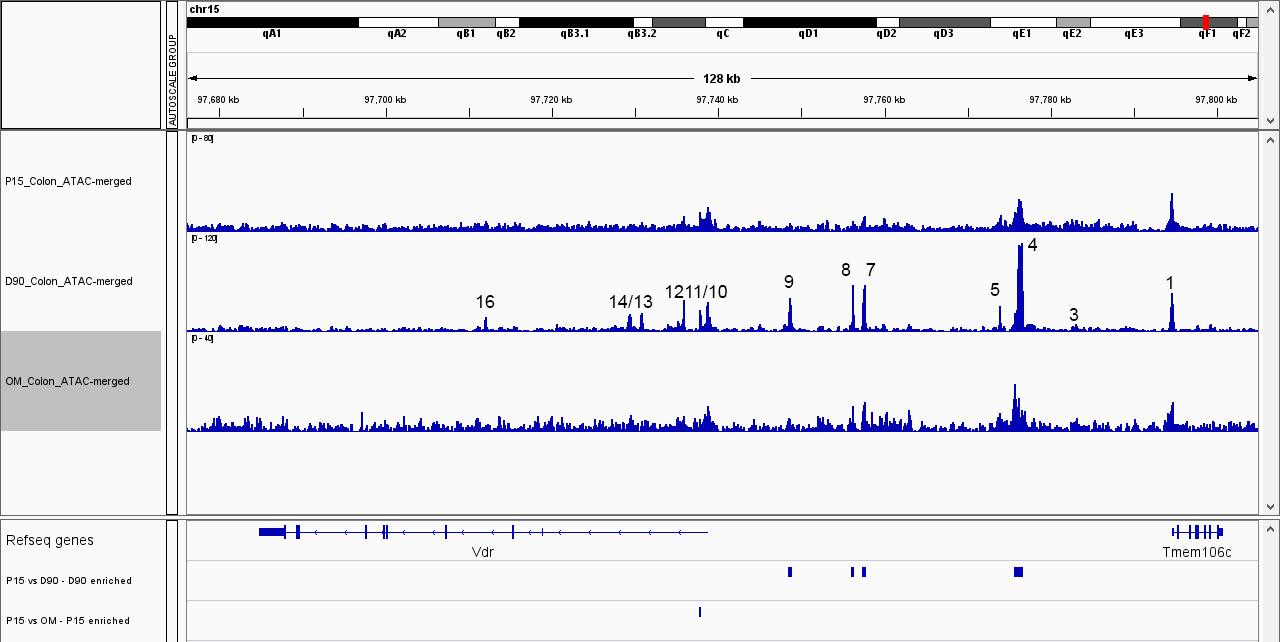


**Supplemental Figure 6S.** Effects of age on ATAC peaks in the colon. OM = old mouse


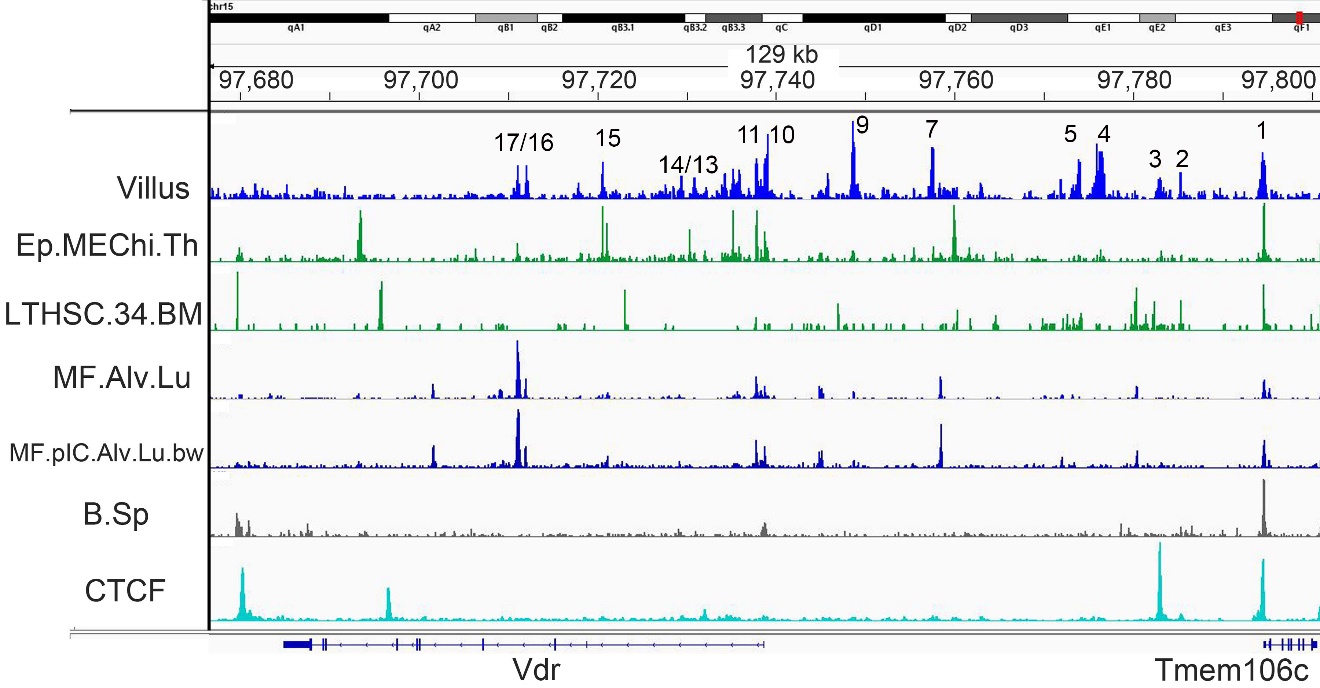


**Supplementary Figure 7S.** Accessible DNA regions controlling mouse Vdr gene expression in small intestine villus compared to various cells of the mouse immune system. Immune system ATAC data is from the Immgen project. Ep.MEChi.Th = thymic epithelial cells; LTHSC.34.BM = stem cells; MF.Av.Lu = lung alveolar macrophages (unstimulated); MF.pIC.Alv.Lu.bw = pIC activated lung alveolar macrophages; B.Sp. = B cells.

**Supplemental Table 1S .** TFBS Predictions from CiiDER analysis of DNA sequences under ATAC Peaks in the mouse intestine. The analysis was conducted with settings at 0.25, 0.2, and 0.15. The presence of ChIP peaks for the transcription factor was presented in the last column.


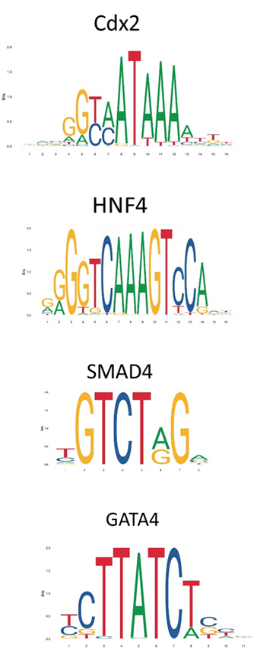

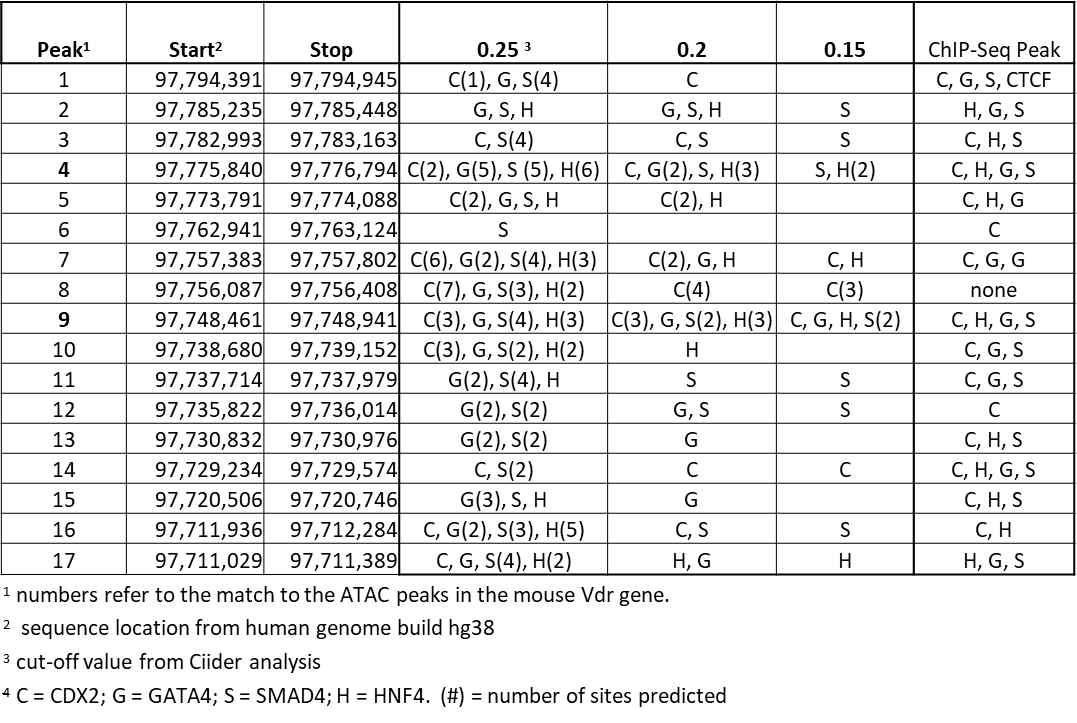


**Supplemental Table 2S .** TFBS Predictions from CiiDER analysis of DNA sequences under DNAse-seq peaks in the human intestine.


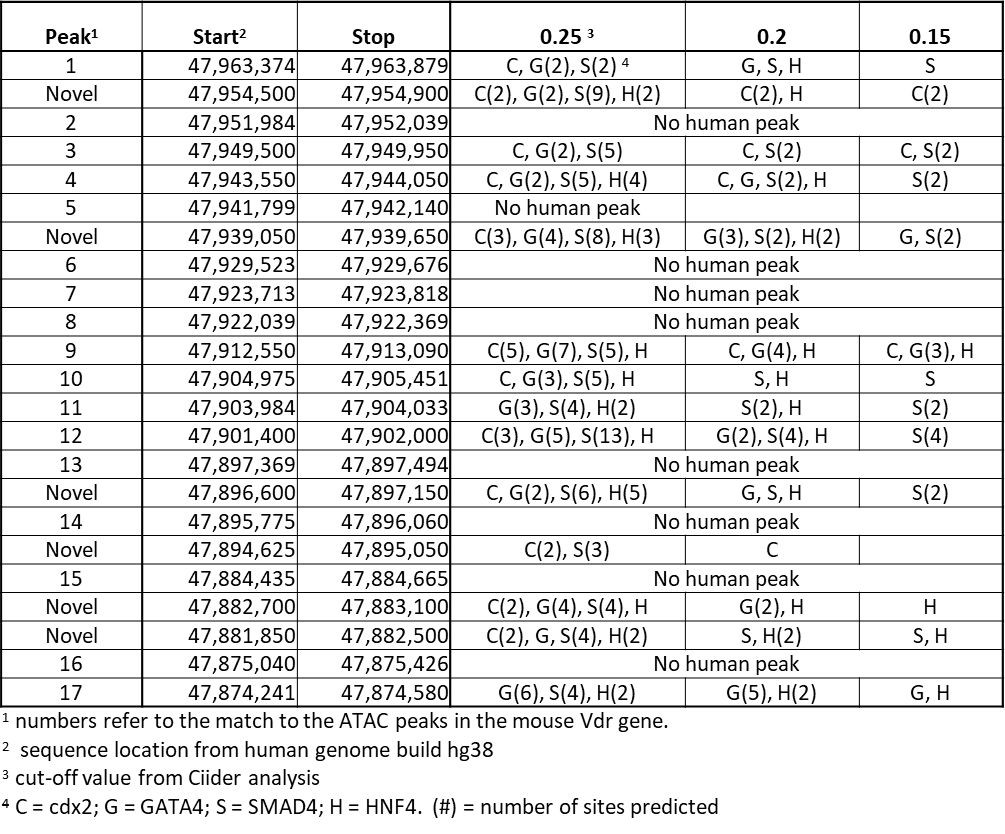

Supplement: Supplemental Figures S1–S7 and Tables S1, S2 [file mmc1.docx]
